# Genetic diversity of Albanian goat breeds revealed by mtDNA sequence variation

**DOI:** 10.1080/13102818.2014.901672

**Published:** 2014-06-04

**Authors:** Anila Hoda, Ylli Biçoku, Petrit Dobi

**Affiliations:** ^a^ Department of Animal Production, Faculty of Agriculture and Environment, Agricultural University of Tirana, Kodër Kamëz, Tirana, Albania

**Keywords:** local goat breeds, mtDNA, genetic diversity

## Abstract

Albanian farmers have a long tradition in goat farming. Recently, several studies were carried out to determine genetic diversity of local goat populations, using molecular markers such as SNP (Single Nucleotide Polymorphisms), microsatellites and AFLP (Amplified Fragment Length Polymorphism). In the present study 77 mtDNA D-loop sequences from six different goat breeds were analysed. The results revealed 67 different haplotypes, with haplotype diversity ranging from 0.864 to 1 and nucleotide diversity values ranging from 0.016 to 0.106. The results showed that the studied breed grouped only in lineage A. The *F*_ST_ analysis indicated that 98.7% of the variation was found within the goat breeds and only 1.3% among them.

## Introduction

Albania has a long tradition in goat breeding. There are several breeds of goats in Albania. These different breeds have different visible and performance characteristic.[1] Six Albanian goat breeds were recently characterized at the molecular level, using several molecular markers such as microsatellites, SNP and AFLP.

The genetic diversity of goat breeds has been studied based on mtDNA sequences by several authors. Naderi et al. [2] reported 1540 haplotypes among 2430 individual animals, including also 77 individuals belonging to six Albanian goat breeds of this present study. Sultana et al. [3] identified 38 haplotypes among 44 individuals from 13 Pakistani goat breeds. Sardina et al. [4] observed 33 haplotypes among 67 individuals of three Sicilian goat breeds. Odahare et al. [5] analysed the mitochondrial DNA diversity of 19 Korean native goats. Pereira et al. [6] traced the history of goat pastoralism in North Africa.

In the present paper the variation of the mtDNA control region in six Albanian goat breeds is analysed. The aim of the study was to analyse the genetic diversity of Albanian goat breeds and to compare the results with those obtained previously by other molecular markers.

## Materials and methods

The 598 bp (base pairs) fragment corresponding to the hypervariable mitochondrial DNA (mtDNA) control region [2] was analysed in 77 individual animals from six Albanian goat breeds (Table 1).

**Table 1.** Values of genetic diversity.

Breed	Number of sequences	Number of haplotypes	Nucleotide diversity	Haplotype diversity	Fu's *Fs* test	Tajima's *D* test
Capore	15	14	0.106	0.990	−0.148*	−2.373***
Dukati	13	13	0.022	1.000	−5.589**	−0.789
Hasi	14	13	0.016	0.989	−5.469**	−0.950
Liqenasi	12	6	0.019	0.864	2.892*	0.0150
Mati	11	10	0.017	0.982	−2.823*	−1.069
Muzhake	12	12	0.017	1.000	−5.85**	−0.987
Total	77	67	0.036	0.996	−47.001***	−2.726***

*P < 0.05; **P < 0.01; ***P < 0.001.

The sequences from GenBank (Accession No. EF617601–EF617677) were edited by using the BioEdit 7.0 software.[7] All sequences of the HVI segment of the mtDNA control region were aligned utilizing the ClustalX package.[8] Gaps in the aligned sequences were excluded from the analyses.

A neighbour-joining (NJ) tree based on the aligned sequences was constructed using the Kimura two-parameter model and a bootstrap value of 10,000, using the MEGA 4 software package.[9] Median-joining networks were calculated using Network 4.2.0.1.[10]

The haplotype diversity (*h*), nucleotide diversity (π) for the breeds and the whole population, average number of nucleotide differences (*D_A_*), and average number of nucleotide substitutions (*Dxy*) per site between breeds were estimated by using the DnaSP 4.1 software.[11] The same software was used to carry out neutrality tests, calculating Tajima's *D* [12] and Fu's *Fs*.[13]

Differentiation between breeds was determined by analysis of molecular variance AMOVA.[14] This analysis and pairwise *F*_ST_ values across goat breeds were computed using Arlequin v.3.1.[15]

## Results and discussion

### mtDNA variation and haplotype in goat breeds

A total of 77 mtDNA D-loop sequences from six different goat breeds were analysed. The analysis showed 67 different haplotypes determined by 15 transitions and 11 transversions, with a transition/transversion ratio of 1.4; as well as two insertion–deletion (indel) mutations. Haplotype diversity was 0.864 for Liqenasi breed and 1 for Dukati and Muzhake. Nucleotide diversity values ranged from 0.016 (Hasi) to 0.106 Capore (Table 1). Haplotype and nucleotide diversity are two indices of great importance for assessing population polymorphism and genetic differentiation. The average nucleotide diversity and haplotype diversity are 0.036 and 0.996, respectively, indicating high genetic diversity.

In Albanian goat breeds the haplotype diversity ranged from 0.864 to 1.000, with an average value of 0.996, and the nucleotide diversity ranged from 0.016 to 0.106, with an average value of 0.036. Sardina [4] indicated that in three Sicilian goat breeds the haplotype diversity was 0.969 and nucleotide diversity was 0.024. Kul and Ertugrul [16] found that the haplotype diversity for five Turkish goat breeds was 0.09982 and the nucleotide diversity was 0.021. Chen et al. [17] indicated that in 18 Chinese goat breeds the level of haplotype diversity for 18 goat breeds ranged from 0.7121 to 0.9804 and the nucleotide diversity ranged from 0.0159 to 0.0490, while for 13 other domestic goat breeds Liu et al. [18] indicated a level of haplotype diversity of 0.9333–1.0000 and nucleotide diversity of 0.006337–0.025194.

At the individual level, 61 haplotypes (92%) were unique, and 5 haplotypes were shared between individuals. At the breed level, all haplotypes were private, except one that was shared between Hasi and Liqenasi breed.

### Phylogenetic tree of the haplotypes

**Figure 1.**
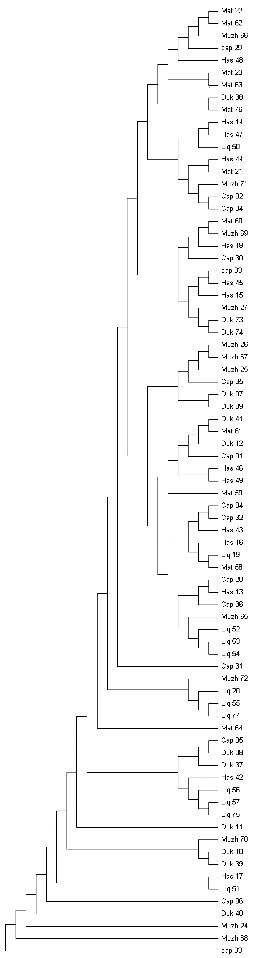
Neighbour-joining bootstrap tree of mtDNA control region sequences of Albanian goat breeds.

The NJ tree for 77 mtDNA haplotypes is presented in Figure 1. Based on the classification of goat mtDNA haplotypes [2] all haplotypes were grouped in haplogroup A. These results indicate the origin of local goat breeds from one population.

**Figure 2.**
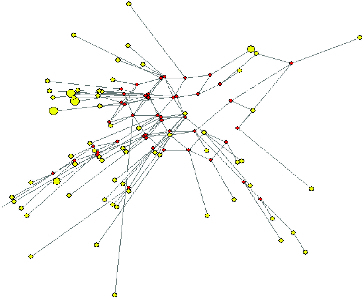
Median-joining network depicting the relationships between mitochondrial haplotypes in the Albanian goat population.

The phylogenetic relationship between Albanian goat haplotypes was inferred using median-joining network analysis (Figure 2). The area of the circles is proportional to the frequency of specimens in the sample. The network topology revealed a substantial divergence among haplotypes, with a large number of independent branches giving rise to multiple sub-branches separated by several mutations.

The *F*_ST_ analysis indicated that 98.7% of the variation was found within the goat breeds and only 1.3% (*P* = 0.039) among them. These results show a low level of breed differentiation and are in concordance with the results obtained previously for the same goat breeds, using microsatellites,[19] SNP [20] and AFLP.[21] Table 2 represents the pairwise *F*_ST_ values among breeds with their significance levels. The pairwise *F*_ST_ values indicate that the values between Liqenasi and three other types (Dukati, Hasi, Mati) were significantly different from zero.

**Table 2.** Pairwise *F*_ST_ values of the six goat breeds based on mtDNA haplotypes.

	Capore	Dukati	Hasi	Liqenasi	Mati	Muzhake
Capore						
Dukati	−0.001					
Hasi	−0.002	0.035				
Liqenasi	0.009	0.049*	0.077**			
Mati	−0.18	0.048	0.013	0.105**		
Muzhake	−0.007	0.008	0.035	0.037	0.066	

*P < 0.05; **P < 0.01.

### Population structure

**Figure 3.**
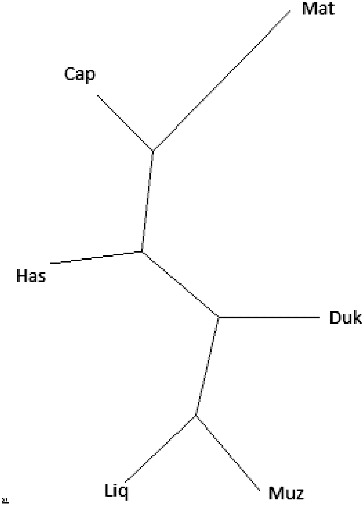
NJ trees for six Albanian goat breeds, based on nucleotide divergence (DA) between populations.

Table 3 shows the estimate of nucleotide divergence between six populations and Figure 2 the NJ tree derived from *D_A_* (average number of nucleotide differences) (Figure 3). The NJ tree shows that the Albanian goat breeds are intermixed. AMOVA analysis (Table 4) showed that 1.3% of the variation is among populations and 98.7% is within populations.

**Table 3.** Matrix of *D_A_* distances (average number of nucleotide differences; above diagonal) and *Dxy* (average number of nucleotide substitutions per site between breeds; below diagonal) among goat breeds.

	Capore	Dukati	Hasi	Liqenasi	Mati	Muzhake
Capore		30.83077	29.32857	30.80000	29.50303	29.62778
Dukati	0.18791		9.27473	10.33333	9.72727	9.26282
Hasi	0.05568	0.31136		9.14286	7.96753	8.08333
Liqenasi	0.67229	0.51515	0.69464		9.75758	8.99306
Mati	−0.04589	0.48788	0.09810	1.03333		8.65152
Muzhake	0.14401	0.08858	0.27905	0.33396	0.57121

**Table 4.** Partitioning of the genetic variance among breeds revealed by hierarchical AMOVA.

Source of variation	d.f. (Degree of freedom)	Sum of squares	Variance components	Percentage of variation
Among populations	5	49.713	0.112Va	1.30
Within populations	71	604.001	8.507Vb	98.70
Total	76	653.714	8.619	

The results from the neutrality tests are given in Table 1. Tajima's *D* value was negative. The data in Table 1 indicate low nucleotide diversity values but high haplotype diversity, which suggests a recent population growth.

**Figure 4.**
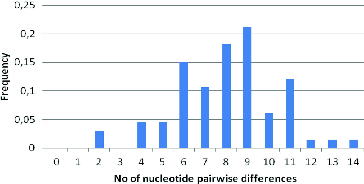
Mismatch distributions for the Albanian goat population.

The mismatch distribution is shown in Figure 4. The mean value observed for the Albanian goat population was 8.79. Based on this distribution, it is suggested that Albanian goat breeds display a constant population size. The value of the sum of squared differences between the observed and the estimated mismatch distribution by a bootstrap approach was 0.333.

In a previous study, Naderi et al. [2] characterized the genetic diversity of domestic goats with 2430 individuals from all over the world, including also 77 individuals from Albania. They showed that these breeds belong to lineage A. This is also confirmed by the phylogenetic tree (Figure 1). We may conclude that Albanian goats have one maternal lineage.

We found a very large negative value of *Fs*. This can be considered as an evidence of population expansion.[13]

## Conclusions

In this study, we observed high mtDNA diversity in Albanian local goat breeds. All the Albanian goats, representing 67 haplotypes, were classified into lineage A, indicating that the origin of local goat breeds is from one population. However, additional molecular studies are required in the near future.
